# Experimental platform utilising melting curve technology for detection of mutations in *Mycobacterium tuberculosis* isolates

**DOI:** 10.1007/s10096-018-3246-2

**Published:** 2018-04-20

**Authors:** Agnieszka Broda, Vlad Nikolayevskyy, Nicki Casali, Huma Khan, Richard Bowker, Gemma Blackwell, Bhakti Patel, James Hume, Waqar Hussain, Francis Drobniewski

**Affiliations:** 1 Infectious Diseases, Department of Medicine, Imperial College London, London, UK; 2 Enigma Diagnostics Ltd, Salisbury, UK

**Keywords:** *Mycobacterium tuberculosis*, Drugs resistance, Molecular assays, Melting curves, Multidrug-resistant tuberculosis

## Abstract

Tuberculosis (TB) remains one of the most deadly infections with approximately a quarter of cases not being identified and/or treated mainly due to a lack of resources. Rapid detection of TB or drug-resistant TB enables timely adequate treatment and is a cornerstone of effective TB management. We evaluated the analytical performance of a single-tube assay for multidrug-resistant TB (MDR-TB) on an experimental platform utilising RT-PCR and melting curve analysis that could potentially be operated as a point-of-care (PoC) test in resource-constrained settings with a high burden of TB. Firstly, we developed and evaluated the prototype MDR-TB assay using specimens extracted from well-characterised TB isolates with a variety of distinct rifampicin and isoniazid resistance conferring mutations and nontuberculous Mycobacteria (NTM) strains. Secondly, we validated the experimental platform using 98 clinical sputum samples from pulmonary TB patients collected in high MDR-TB settings. The sensitivity of the platform for TB detection in clinical specimens was 75% for smear-negative and 92.6% for smear-positive sputum samples. The sensitivity of detection for rifampicin and isoniazid resistance was 88.9 and 96.0% and specificity was 87.5 and 100%, respectively. Observed limitations in sensitivity and specificity could be resolved by adjusting the sample preparation methodology and melting curve recognition algorithm. Overall technology could be considered a promising PoC methodology especially in resource-constrained settings based on its combined accuracy, convenience, simplicity, speed, and cost characteristics.

**Electronic supplementary material:**

The online version of this article (10.1007/s10096-018-3246-2) contains supplementary material, which is available to authorized users.

## Introduction

Tuberculosis (TB) has re-emerged as the leading cause of mortality associated with an infectious disease globally causing 1.8 million deaths from an estimated 10.4 million incident of TB cases in 2015 [1]. Of these TB cases, 11% (including 0.4 million deaths) occur in HIV co-infected patients [2]. The mainstay of diagnosis in most global settings remains sputum smear microscopy which lacks both sensitivity and specificity [3, 4]. Patients, particularly children, frequently cannot freely expectorate sputum, and HIV co-infected patients produce lower TB bacterial numbers in their sputum. There remains a significant need for new rapid diagnostic systems.

More recently nucleic acid amplification (NAAT) systems such as line probe assays (LPAs) (sensitivity 86.7–100% and specificity 82.4–100%) [5] and the Xpert® MTB/RIF machine and assay (Cepheid Inc., Sunnyvale, CA, USA) (sensitivity 97.6% and specificity 99.2%) [6] have been implemented approaching microbiological culture in terms of sensitivity and specificity but offering significantly shorter turnaround times.

Innovative assays based on high-resolution melting curve analysis have been developed recently and evaluated for rapid detection of TB and rifampicin and isoniazid resistance. In these assays, fluorescent probes bind to specific gene sequences in a temperature-dependent order and measured fluorescence rises and falls as temperature increases allowing even a single-nucleotide mismatch to be detected by a characteristic fluorescence signature.

In our study, we evaluated the analytical performance of a novel assay and an experimental platform for testing multidrug-resistant tuberculosis (MDR-TB), resistant to at least rifampicin (RIF) and isoniazid (INH) that could potentially be operated as a point-of-care (PoC) test. The assay is based on a novel highly multiplexed DNA-sensing technology and melt curve analysis utilising an innovative magnetic bead extraction methodology for isolation of TB cells from sputum [7]. These have been integrated into a single-use cartridge for use within the platform which has been designed for direct processing of clinical samples without the need for user intervention.

## Materials and methods

### Study design

The study had two major phases. The first phase of the study was a development and evaluation of the performance of the prototype MDR-TB wet assay using the LightCycler® 2.0 instrument (Roche Diagnostics GmbH, Mannheim, Germany) comprising 120 DNA specimens (panel 1) followed by a blind validation of a prototype freeze-dried assay using a second panel (panel 2).

The second phase was a validation of the experimental platform technology on 98 clinical sputum samples comprising panel 3.

### Test panels

Panel 1 included a total of 120 specimens of purified genomic DNA (gDNA) samples from *Mycobacterium tuberculosis* complex (Mtbc) (*N* = 91) and non-purified DNA from nontuberculous Mycobacteria (NTM) cultures (*N* = 29) (Table S1). It included duplicate samples to assess reproducibility and sample-to-sample and run-to-run variation. Within the panels, Mtbc and NTMs were identified using line probe assays (GenoType® Mycobacterium CM assay, GenoType® Mycobacterium MTBC assay, and GenoType® AS assay (Hain Lifescience GmbH, Nehren, Germany)). NTM species were additionally confirmed using Sanger sequencing of 16s RNA genes. Mutations in *rpoB*, *katG*, and the promoter region of *inhA* genes were characterised by whole genome sequencing (WGS).

RIF- and INH-resistant *M. tuberculosis* isolates within this panel (*N* = 80) harboured 50 distinct common and rare *rpoB* mutations (single, double, and triple SNPs, as well as several small non-frame shifting indels), 4 *katG* variants, and 4 *inhA* promoter polymorphisms (Tables S2 and S3).

Panel 2 contained purified gDNA samples extracted from RIF- and INH-sensitive and RIF- and INH-resistant clinical TB isolates, including 70 *Mycobacterium tuberculosis* isolates, 10 positive controls (*M. tuberculosis* H37Rv), and 10 Mtbc species (*M. bovis*, *M. africanum*, *M*. *canetti*, *M*. *microt*i, *M*. *pinnipedii*), and 29 samples of nontuberculous Mycobacteria species were included in panel 1.

Panel 3 consisted of 98 consecutive sputum specimens collected from 98 hospitalised patients (aged ≥ 18), with pulmonary TB, at the Infectious Diseases and Tuberculosis Hospital in Vilnius, Lithuania. Residual sputum specimens (leftover from specimens collected as part of their routine clinical management) were collected consecutively between September and November 2015 until the planned number of smear positive and negative was achieved. Among 98 collected sputum specimens, 75 were smear positive and 23 were smear negative with 61 and 8 culture-positive specimens within these groups, respectively. Specimens were collected before a treatment commenced. Anonymised residual sputum specimens were tested at the local hospital using a range of molecular and phenotypic reference assays (see below).

### Specimen processing for assay development and evaluation

#### Bacterial culture and DNA isolation (panels 1 and 2)

Isolates were cultured on Middlebrook 7H9 broth for 2 to 4 weeks (NTMs) at 30–37 °C or on Middlebrook 7H11+OACD agar plates for 4–6 weeks (Mtbc) at 37 °C. Sweeps of Mtbc colonies were harvested, resuspended in TE, and inactivated by heating at 80 °C for 50 min. After lysis by vortexing for 3 min with 0.1-mm glass beads, gDNA was purified using a DNeasy® Blood and Tissue Kit (QIAGEN GmbH, Hilden, Germany). The purified gDNA was used for PCR reactions. Alternatively, NTM colonies were resuspended in TE, mixed with an equal volume of chloroform, and heated at 80 °C for 50 min. This inactivated and lysed bacteria (non-purified DNA) was used with PCR mix.

#### Sputum specimens processing (panel 3)

The chemistry of sample preparation was based on a proprietary magnetic bead extraction technology (Microsens Diagnostics Ltd., London, UK): 5 ml of sputum samples was mixed with two volumes of a deactivation reagent (20% isopropanol, 2 M sodium hydroxide) and incubated for 15 min. Effective inactivation of *M*. *tuberculosis* bacteria was demonstrated using in-house viability checks before incorporating the extraction technology into the prototype platform.

Approximately 1.5 ml of decontaminated and liquefied sputum was transferred to a cartridge tube. The cartridge was inserted into the prototype instrument (run time approximately 2 h).

#### MDR-TB assay design

The MDR-TB assay was designed for detection of *M. tuberculosis*, differentiation between members of the *M. tuberculosis* complex (via detection of gyrB sequence differences), and detection of mutations in the *rpoB*, *katG*, and *inhA* genes associated with resistance to RIF and INH on primary sputum specimens. The MDR-TB assay amplifies and detects four targets in the Mtbc genome (*rpoB*, *gyrB*, *katG*, *inhA*) and *Lactococcus lactis cremoris* (process control for assay amplification and contamination) using four fluorophores in four optical channels (Table S4).

Development and initial evaluation of the MDR-TB assay was performed with panel 1. A validation was performed with panel 2 to determine assay analytical sensitivity and specificity using the LightCycler® 2.0 instrument (Roche Diagnostics GmbH, Nehren, Germany). PCR reactions were carried out in a final reaction volume of 20 μl (Table S5). Samples were amplified as follows: initial denaturation step at 95 °C for 3 min; 60 cycles of denaturation at 95 °C for 10 s, annealing at 68 °C for 25 s, and extension at 72 °C for 20 s followed by 95 °C 30 s; 40 °C 30 s; and ramping to 90 °C with continuous melt curve data acquisition. Total genomic DNA from H37Rv reference TB strain and nuclease-free DEPC-treated water were used as positive and negative controls, respectively. Primer and probe sequences are given in Table S6.

#### Freeze-dried MDR-TB PCR assay

For each reaction, 2 μl sample and 2 μl genomic *Lactococcus* DNA were combined with 36 μl nuclease-free DEPC-treated water and used to resuspend one freeze-dried assay cake, a lyophilised mixture containing optimised target-specific primers, buffers, salts, and enzyme. The cake was allowed to rehydrate for 1 min and mixed by pipetting. The 20 μl was transferred to a capillary and reactions loaded onto the LightCycler 2.0.

### Reference tests

#### Phenotypic and molecular characterisation of sputum specimens (panel 3)

Reference diagnostic tests were performed on anonymised residual sputum specimens at the TB Reference Laboratory, Infectious Diseases and Tuberculosis Hospital, affiliate of Public Institution Vilnius University Hospital Santariskiu Klinikos. Specimens were processed following the standard NALC-NaOH method [8]. Concentrated sediment was resuspended in phosphate buffer and inoculated onto automated Bactec MGIT960 system tubes and LJ (Lowenstein Jensen) slants. Graded sputum smear microscopy was performed by ZN (Ziehl-Neelsen) staining [8]. Drug susceptibility testing for RIF and INH was performed on all positive cultures using the MGIT960 system (Becton Dickinson, Oxford, UK) with standardised drug concentrations [9]. DNA for GenoType assays (Hain Lifescience, Nehren, Germany) was extracted from an aliquot of resuspended sample pellet by heating for 20 min at 95 °C, followed by incubation for 15 min in an ultrasonic bath. The GenoType MTBDRplus assay was carried out as described by the manufacturer.

### Ethical permission

Ethical approval for the collection of residual sputum specimens was obtained at the Infectious Diseases and Tuberculosis Hospital in Vilnius, Lithuania. Ethical approval for the whole study was received from the Imperial College London (ICL) Research Ethics Committee. Patients invited to take part in the study were provided with an information sheet and informed consent was obtained before the enrolment into the study. No patient data was recorded. All specimens were fully anonymised and personal identifiers removed so specimens could not be traced back to patients.

### Data analysis

Specimen processing and data analysis for both panels 2 and 3 were performed by operators in a blinded manner with no access to reference test results.

The raw data for panel 3 generated at ICL was sent for blinded analysis using proprietary algorithms. Results (i.e. TB, NTM, isoniazid and rifampicin resistance or sensitivity) were returned to ICL and performance was compared to reference diagnostic tests. Initial data analysis and performance characteristic calculations (sensitivity, specificity, NPV, and PPV) were performed using Microsoft Excel 2010.

## Results

### Initial evaluation of MDR-TB assay on the LightCycler 2.0 instrument

Development and initial validation of the MDR-TB assay was performed using the wet assay format on panel 1. Primers, probes, fluorophores, and their combinations were optimised to ensure optimal discrimination of all mutants from wildtype strains, identification of *M*. *tuberculosis* and *M. bovis*, and distinguishing them from NTMs. Probes and primers were selected and optimised, and an algorithm for identification of Mtbc species and detection of mutations in gyrB, *rpoB*, *katG*, and *inhA* genes using melting curve profiles (Figs. S1, S2, S3, and S4) was developed and subsequently incorporated into the MDR-TB assay analytical pipeline.

### Blinded validation of MDR-TB prototype assay on the LightCycler RT-PCR system

The MDR-TB prototype assay developed on panel 1 was subsequently validated on panel 2 using the LightCycler 2.0, the testbed for the assay prototype platform. Two independent operators visually inspected melting curves and distinguished between wildtype and mutant variants of *rpoB*, *katG*, and *inhA* genes for Mtbc.

Sensitivity and specificity for Mtbc identification and detection of mutations of the prototype assay compared to WGS based on visual inspection of melting curves were ranging from 98.6 to 100% (Table 1). Both operators were in agreement in all but one case of Mtbc versus NTM detection; this was marked as ambiguous result and excluded from further calculations. Specificity and sensitivity for *M*. *tuberculosis* and *M. bovis* detection were 100%. Sensitivity for *rpoB* and *katG* mutant detection was 98.6% for both genes; the prototype assay missed the *rpoB* H445D mutation and one *katG* mutation. Specificity was 100% for all targets.

**Table 1:** Performance characteristics of the MDR-TB wet assay, with panel 2 samples, run on the LightCycler instrument and visual interpretation of melting curve profiles

Target	Sensitivity (%)	Specificity (%)
*gyrB:* for Mtbc detection	100.0	100.0
*gyrB:* for distinguishing between *M. tuberculosis* and *M. bovis*	100.0	100.0
*rpoB*	98.6	100.0
*katG*	98.6	100.0
*InhA*	100.0	100.0

### Evaluation of the experimental MDR-TB platform

Performance of the platform on panel 3 was assessed separately for smear-positive and smear-negative sputum specimens versus GenoType MTBDRplus reference test results. Interpretable results for the experimental MDR-TB assay were obtained for 87 specimens (88.8%). No results (due to an absence of internal control amplification) were recorded for 11 specimens (11.2%); these were excluded from further analysis.

The sensitivity of the prototype platform for TB detection in primary specimens was 75 and 92.6% for smear-negative and smear-positive samples respectively (Table 2). The specificity was 83.3% for smear-negative samples. The specificity for smear-positive samples could not be calculated. Among 69 smear-positive samples, one was negative by GenoType MTBDRplus and five by the MDR-TB assay but none of the negative results were negative by both tests at the same time so the specificity was non-calculable.

**Table 2:** Performance characteristics of MDR-TB platform for Mtbc and RIF and INH resistance detection on primary sputum specimens calculated using GenoType MTBDRplus as a blinded reference test (*n* = 87) (panel 3)

MDR-TB platform	Mtbc detection		RIF and INH resistance detection	
	Smear −ve	Smear +ve	RIF	INH
Sensitivity	9/12 (75%)	63/68 (92.6%)	40/45 (88.9%)	48/50 (96%)
Specificity	5/6 (83.3%)	0/1 (non-calculable)	21/24 (87.5%)	22/22 (100%)
PPV	90%	100%	93%	100%
NPV	61.50%	Non-calculable	80.80%	91.70%

One of the specimens was negative (no amplification) by GenoType MTBDRplus but was identified as *M. bovis* by the experimental assay. The sensitivity of the LightCycler 2.0 MDR-TB assay for rifampicin and isoniazid resistance was 88.9 and 96.0%, respectively, and specificity was 87.5 and 100%, respectively (Table 2).

Analysis of discrepancies in drug susceptibility testing results between tested platform and GenoType MTBDRplus revealed the presence of both false-positive and false-negative results. Polymorphisms missed in resistant isolates included the most common mutations S315T in *katG* and S531L in *rpoB* genes (Table 3).

**Table 3:** Discrepancies in isoniazid (A) and rifampicin (B) susceptibility test results in sputum samples. GenoType MTBDRplus versus MDR-TB platform (R-resistant; S-sensitive) (panel 3)

A				
Isolates	Isoniazid			
	GenoType MTBDRplus		MDR-TB platform	
	katG	inhA	katG	inhA
V002	R (S315T)	R (C15T)	R	S
V023	R (S315T)	S	S	S
V052	R (S315T)	S	R	R
V064	R (S315T)	S	S	S
B				
Isolates	Rifampicin			
	Hain GT	MDR-TB platform		
V023	R (S531L)	S		
V039	R (S531L)	S		
V052	R (S531L)	S		
V056	R (S531L)	S		
V064	R (S531L)	S		
V075	S	R		
V085	S	R		
V093	S	R		

A separate analysis of the assay’s performance for TB and RIF and INH resistance detection conducted using MGIT culture results as a reference standard demonstrated similar sensitivity but lower specificity (Table S7 and S8).

## Discussion

We assessed analytical performance of a prototype MDR-TB assay and the experimental prototype platform for rapid detection of *M*. *tuberculosis* and *M. bovis* and identification of resistance to key first-line drugs (RIF and INH) in clinical respiratory specimens.

Several commercial diagnostic assays for detection of Mtbc and resistance to key anti-TB drugs based on nucleic acid amplification technology (NAAT) are currently in use in diagnostic laboratories [4]. The GeneXpert test endorsed by WHO was designed to be a near point-of-care (PoC) device [9]. Validation studies acknowledged good performance characteristics of many TB assays (such as Hain Genotype MTBDRPlus/CM/AS, GeneXpert, and INNO-LiPA RifTB) but noted that further work was required to improve sensitivity on primary specimens and address other issues including heteroresistance and cross-reactivity resulting in false-positive and false-negative results for TB and drug resistance detection [10, 11].

Studies with the GeneXpert platform reported cross-reactivity with NTM strains which resulted in false-positive rifampicin resistance due to early Ct values by cross-hybridisation with NTM [12]. This indicates a need for developing an assay which is based on RT-PCR coupled with melting curve analysis, specifically high-resolution melting curve analysis (HRMA) eliminating downstream processing of PCR products (such as the hybridisation of mutants to a membrane strip used by GT Blot 20 or 48 machine (Hain Lifescience) or gel electrophoresis) and cross-contamination from amplicons by using the closed tube system [13].

The newest assays developed to address this issue such as the lab-on-chip-based platform (ST Microelectronics, Geneva, Switzerland), a molecular assay designed for detection of MDR-TB in low-income countries with an accuracy of 97.8% [14], and Xpert MTB/RIF Ultra, the upgraded version of GeneXpert recommended by WHO for children and extra-pulmonary and HIV co-infected patients utilising melt curve analysis [15]. The TB-LAMP, another closed system assay recommended as an alternative for smear microscopy in countries with an intermediate or high TB burden, has a lower sensitivity compared to GeneXpert MTB/RIF [15, 16].

The prototype platform perfectly fits into this group of rapid diagnostic tests with great potential as a PoC device based on accuracy, convenience, simplicity, and speed. Importantly, proposed platform uses the closed system meaning that reaction is performed in one single tube sealed upon transferring an original sputum into it. The whole process does not involve steps like DNA extraction, PCR amplification, and hybridisation and is not prone to amplicon contamination.

The sensitivity of the platform for smear-negative, culture-positive samples was 75% and for smear-positive, culture-positive samples was 92.6% which is slightly lower compared to GeneXpert performance reported by Boehme (76.9 and 98.3%, respectively) [17].

The sensitivity (88.9 and 96.0%) and specificity (87.5 and 100%) for RIF and INH resistance, respectively, compared to GenoType MTBDRplus assay were similar to sensitivities and specificities reported for GenoType MTBDRplus assay and slightly lower than those for INNO-LiPA RifTB and GeneXpert® MTB/RIF (for RIF only) in a recent systematic review [5]. All missed cases were associated with the most common resistant genotypes S450L and S315T in *rpoB* and *katG* genes respectively. These mutations are found in 90% of Beijing isolates and 67% of Euro-American isolates and in 74% of Beijing and 30% of Euro-American isolates, respectively [18]. However, the same wet prototype assay, performed on LightCycler 2.0 and assessed visually, gave an optimal performance with 100% specificity and sensitivity for detection of Mtbc and differentiation between Mtbc and NTMs. The high sensitivity and specificity were also achieved for detection of *rpoB* and *katG* mutations (Table 1). Thus, the suboptimal performance of the platform for INH and RIF resistance detection indicates that more work on the chemistry of sample preparation and on the melting curve reading algorithm is required. This problem was also indicated by the proportion of unreadable results (11%). It was significantly higher than the 2.4–5.9% observed in studies that reported the performance of GeneXpert [6, 17, 19].

The sample preparation protocol required 5 min hands-on work and minimum involvement and training time of a technician (nurse level). The disinfectant reagent is ready to use and the only time when the technician is potentially exposed to aerosolised organism is when the disinfectant is added to the sputum collection cup. The proposed technology does not require containment level 3 or biosafety cabinets.

Time required to analyse one to four samples (maximum) from the moment of placing the patient’s sample with a technician to receiving the result was 2 h 20 min. The hands-on time was 20 min (including 15 min incubation time) compared to 44 min of GeneXpert, 45 min of INNO-LiPA, and 50 min of GenoType MTBDRplus [5, 20].

Despite some limitations in sensitivity and specificity, which might be solved by adjusting the melting curve algorithm further, the study demonstrated that the platform might be a good alternative to already existing tests.

## Authors’ conclusion

There is a number of tests available for rapid detection of drug-resistant TB. From the health economic perspective, test must be accurate and available at the lowest possible cost that permits commercial success. Thus, different test configurations may be of value to TB services and programmes in countries with different incomes, health delivery systems, and geography. For many middle-income countries, there is a health structure that better suits a more centralised diagnostic approach, whilst for others, a highly disseminated approach would be more appreciate. Different tests are needed for different environments. We do not claim that this technology is superior to the existing ones. We would rather like to see it among a great number of successful tests of different configurations helping to drive down the diagnostic costs and identify the best treatment options for TB patients.

## Electronic supplementary material

ESM 1 (XLSX 25 kb)

ESM 2 (PDF 587 kb)
