# Supplementary material for: Experimental platform utilising melting curve technology for detection of mutations in Mycobacterium tuberculosis isolates
Source: Eur J Clin Microbiol Infect Dis. 2018 Apr 20;37(7):1273–9. doi: 10.1007/s10096-018-3246-2 (PMC6015100; doi:10.1007/s10096-018-3246-2)
Supplement: Supplementary file 2 — (PDF 587 kb) [file 10096_2018_3246_MOESM2_ESM.pdf]

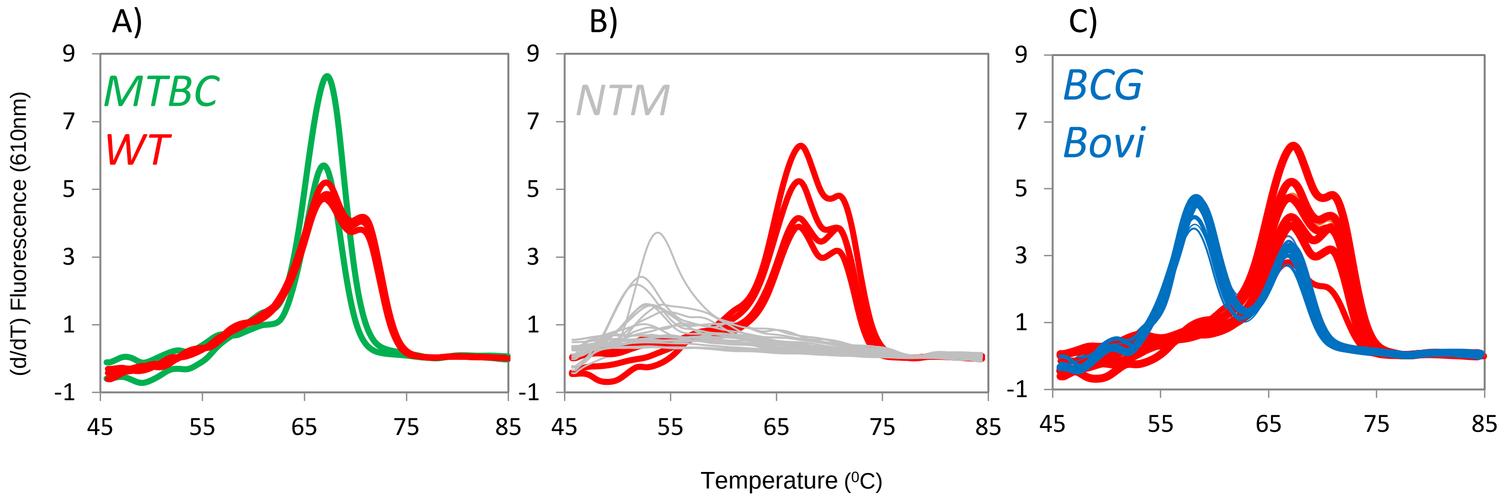

Figure S1. Melting curve profiles for *gyrB* amplicons using the finalised MDR-TB wet assay. A) Wild type (WT-H37Rv)(red) ; Mycobacteria tuberculosis complex (MTBC) including: *M. caprae, M. cannetti, M.africanum, M. microti, M. pinnipedii (gren)* B) Wild type (WT) - H37Rv (red); Nontuberculous Mycobacteria (NTM) (grey) (see table S1) C) Wild type (WT) - H37Rv (red); Bacillus Calmette-Guerin (BCG). *M. bovis* (blue).

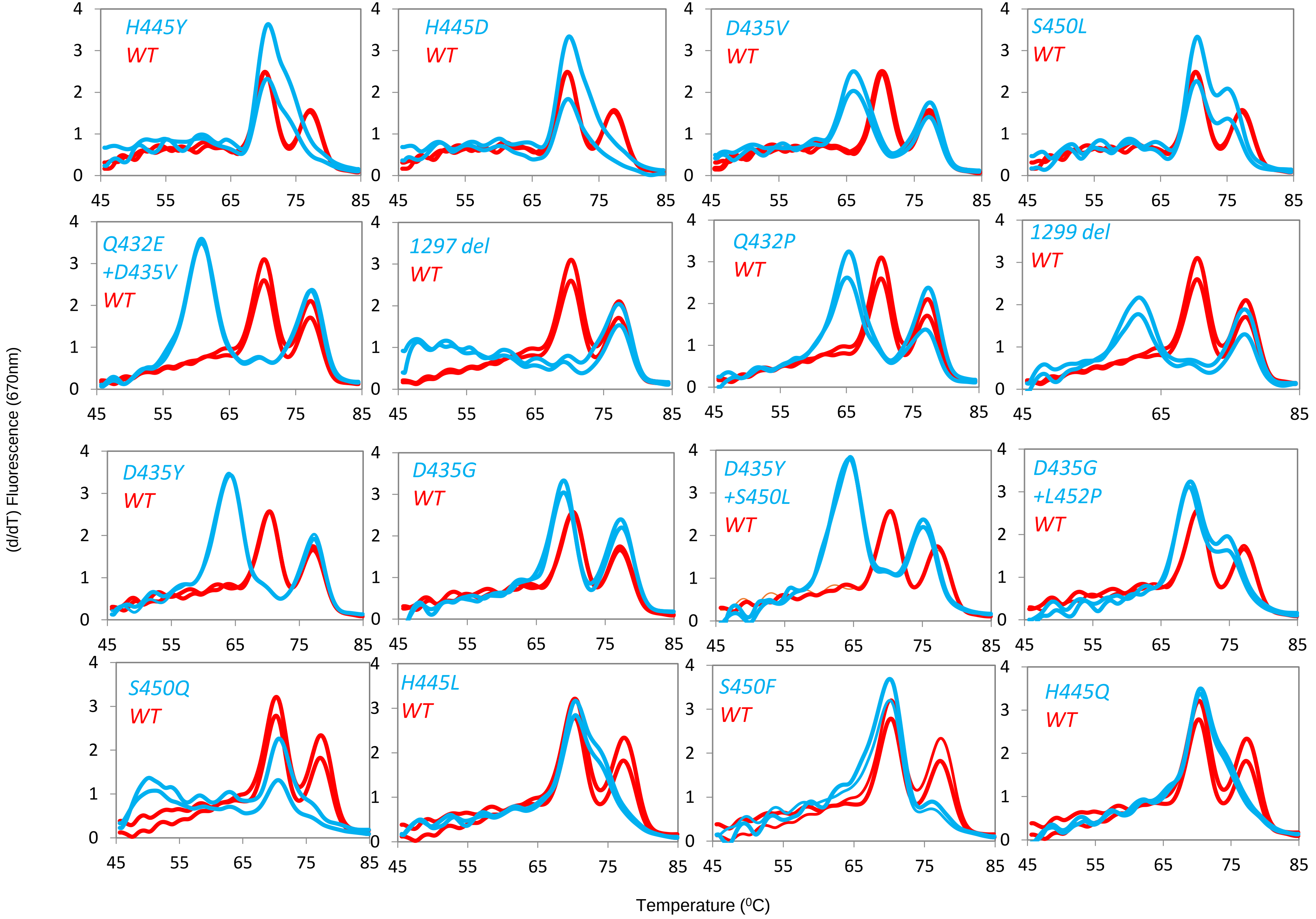

Figure S2. Melting profiles for rpoB mutants (blue) and Wild Type (WT) – H37Rv (red) using finalised MDR-TB wet assay.

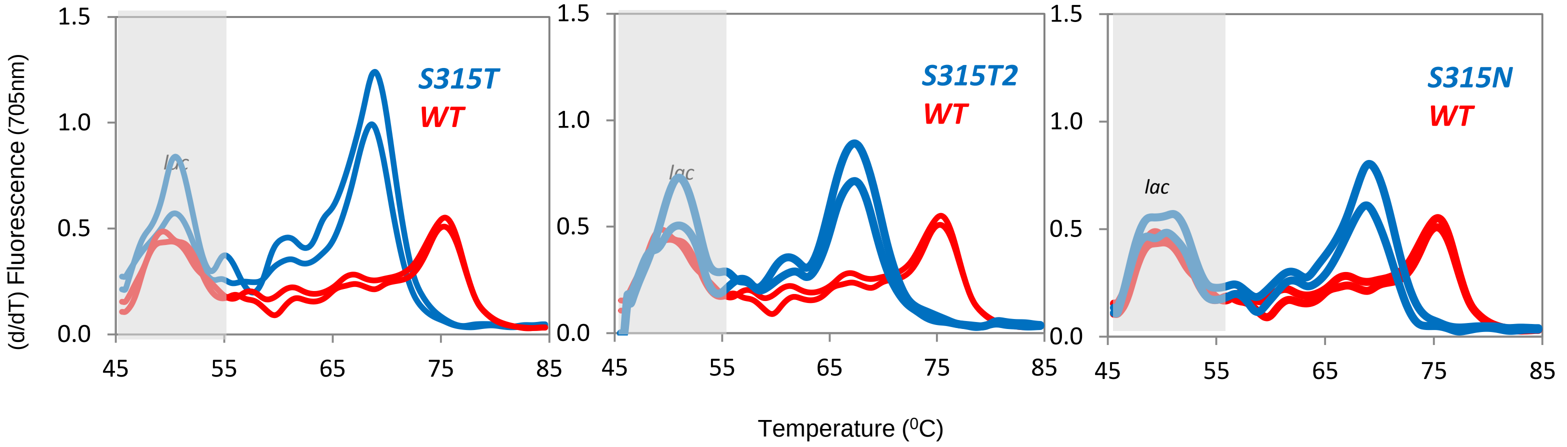

Figure S3. Melting profiles for katG mutants: S315T, S315T2, S315N (blue), Lactococcus amplicons (lac) (blue) and Wild Type (WT) – H37Rv (red) using finalised MDR-TB wet assay.

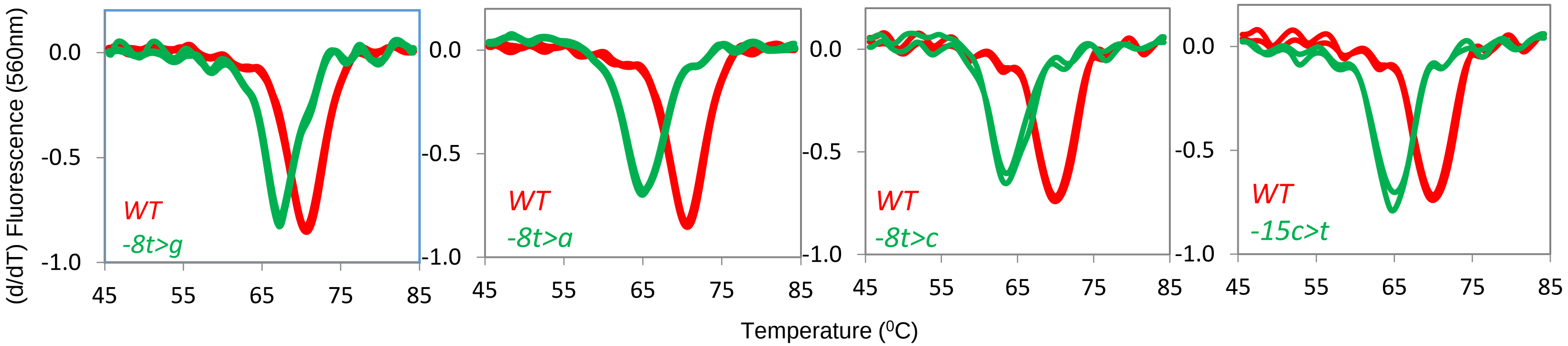

Figure S4. Melting profiles for inhA using finalised MDR-TB wet assay showing inhA mutants: -8t>g, -8t>a, -8t>c, -15c>t (green) and Wild type (WT) –H37Rv (red).
